# Diagnostic radiation procedures and risk of prostate cancer

**DOI:** 10.1038/sj.bjc.6604370

**Published:** 2008-05-13

**Authors:** P Myles, S Evans, A Lophatananon, P Dimitropoulou, D Easton, T Key, R Pocock, D Dearnaley, M Guy, S Edwards, L O'Brien, B Gehr-Swain, A Hall, R Wilkinson, R Eeles, K Muir

**Affiliations:** 1 Division of Epidemiology and Public Health, University of Nottingham Medical School, Queens Medical Centre, Nottingham NG7 2UH, UK; 2 The Royal Marsden NHS Foundation Trust, Fulham Road, SW3 6JJ, UK; 3 CR-UK Genetic Epidemiology Unit, Strangeways Research Laboratories, Worts Causeway, Cambridge CB1 8RN, UK; 4 Cancer Research UK Epidemiology Unit, University of Oxford, Richard Doll Building, Roosevelt Drive, Oxford OX3 7LE, UK; 5 BAUS Section of Oncology, London WC2A 3PE, UK; 6 The Institute of Cancer Research, Sutton, Surrey SM2 5NG, UK; 7 The Chulabhorn Cancer Hospital, Bangkok, 10210, Thailand; 8 University Department of Oncology, Addenbrooke's Hospital, Cambridge CB2 0QQ, UK

**Keywords:** prostate, radiation, case–control, epidemiology

## Abstract

Exposure to ionising radiation is an established risk factor for many cancers. We conducted a case–control study to investigate whether exposure to low dose ionisation radiation from diagnostic x-ray procedures could be established as a risk factor for prostate cancer. In all 431 young-onset prostate cancer cases and 409 controls frequency matched by age were included. Exposures to barium meal, barium enema, hip x-rays, leg x-rays and intravenous pyelogram (IVP) were considered. Exposures to barium enema (adjusted odds ratio (OR) 2.06, 95% confidence interval (CI) 1.01–4.20) and hip x-rays (adjusted OR 2.23, 95% CI 1.42–3.49) at least 5 years before diagnosis were significantly associated with increased prostate cancer. For those with a family history of cancer, exposures to hip x-rays dating 10 or 20 years before diagnosis were associated with a significantly increased risk of prostate cancer: adjusted OR 5.01, 95% CI 1.64–15.31 and adjusted OR 14.23, 95% CI 1.83–110.74, respectively. Our findings show that exposure of the prostate gland to diagnostic radiological procedures may be associated with increased cancer risk. This effect seems to be modified by a positive family history of cancer suggesting that genetic factors may play a role in this risk association.

Prostate cancer (PC) is the most common late-onset cancer in males in developed Western countries (Selley *et al*, 1997). Compared with other common cancers like breast cancer, the aetiology of prostate cancer is not well understood (Gronberg, 2003). Studies in the past have looked at the association between occupational exposure to radiation and an increased risk of prostate cancer and provided conflicting evidence (Beral *et al*, 1985; Atkinson *et al*, 2004). It is interesting, however, that in our literature review we found no published papers on the possible link between exposures to diagnostic radiological procedures and increased prostate cancer risk.

We therefore investigated if there is any association between exposure to low dose ionisation radiation from diagnostic radiological procedures and an increased risk of prostate cancer. Our study focussed on young men with prostate cancer (young onset PC or YOPC) in whom genetic susceptibility may play a role in the incidence and progression of the disease. We conducted a case–control analysis of exposure to five common radiological procedures: barium meal, barium enema, intra-venous pyelogram (IVP), upper leg x-rays and hip/pelvic x-rays, each of which would deliver radiation dose in the region of the prostate. We looked at exposures dating at least 5 years, 10 years and 20 years before the diagnosis of prostate cancer to exclude bias from exposures related to diagnostic and therapeutic procedures for the disease.

## METHODS

### Study participants and data collection

Our study population comprised 431 cases and 409 frequency matched controls from the United Kingdom (UK). Recruitment of cases (to The UK Genetic Prostate Cancer Study; UKGPCS) was through notification by consultants and selected referral centres to the Institute of Cancer Research and The Royal Marsden NHS Foundation Trust. Cases were 60 years or younger and lived in the UK. For each case, controls were recruited from general practice surgeries where the case was registered. Controls were frequency matched to cases by age within 5 years on either side.

Subjects were sent a postal questionnaire including questions on demographic characteristics, lifestyle, occupational exposures and history of diagnostic radiology procedures. The study was approved by the multiregional ethics committee and loco-regional ethical approval was sought where necessary. For this case–control analysis we used data collected on history of barium meal, barium enema, IVP, upper leg x-rays and hip/pelvic x-rays.

### Data analysis

An unconditional logistic regression was performed in SPSS version 11.5. Unadjusted odds ratios (OR) and 95 percent confidence intervals (95% CI) were calculated for each of the exposure variables. Adjustment was done for age at diagnosis of prostate cancer (Gronberg, 2003) and social class based on occupation. We did not adjust for smoking as there is little evidence to suggest it is a confounder for prostate cancer and this was further confirmed in our own analyses. *P*-values were calculated using the *χ*^2^ test for categorical exposure variables. Approximately five percent of the data had missing values and have been excluded from the analysis.

Since some radiological procedures are more common than others such as hip and leg x-ray as compared to barium enema, the study power was calculated based on the exposure rates in controls ranging from 5 to 20 percent. In all 800 subjects (400 cases and 400 controls) would provide 80 percent study power to detect risks (significant at the 5 percent level) of 2.2, 1.8 and 1.6 when exposure rate in controls is 5, 10 and 20 percent respectively. Similarly, the study was powered at 80 percent to detect protective risks of 0.2, 0.3 and 0.4 with exposure rates of 5, 10 and 20 percent respectively in controls(Dupont and Plummer, 1990).

Our original dataset did not have information on dates of exposure for all the radiological procedures undergone by subjects. Subjects instead had been asked for age at first exposure to various radiological procedures. We chose therefore to restrict further analyses to those subjects who had only one procedure and considered exposures at the following time periods: at least 5, 10 and 20 years before diagnosis. A separate analysis was also done for those with a family history of any cancer, which was defined as cancer in any first or second degree relative at any age.

The aetiologic fraction was also calculated using the Meittinen formula (Meittinen, 1974) both with and without controlling for a family history of cancer. We also estimated typical doses provided to the prostate gland from diagnostic x-ray examinations. We used doses either to the urinary bladder or gonads (female) from radiological exposures as a proxy for the dose to the prostate (Hart and Hillier, 1996; Walley and Mantgani, 1997; Ruiz-Cruces and Ruiz, 2000; Hart and Wall, 2002). In some cases the dose was estimated from the incident skin dose using EffDose v.1.0 (which uses data taken from the NRPB report on effective doses from diagnostic radiation (Hart *et al*, 1994)).

## RESULTS

Baseline demographic characteristics of cases and controls are presented in Table 1.

### All subjects

Table 2 summarises the results of our case–control analysis of all subjects irrespective of family history of cancer. The univariate analysis found a significantly increased risk of prostate cancer for the following diagnostic procedures: barium enema and hip/pelvic x-rays, when exposures at least 5 years before diagnosis were considered. Even after adjustment for age and social class, odds ratios remained significantly increased: barium enema OR 2.06, 95% CI 1.01–4.20 and hip/pelvic x-rays OR 2.23, 95% CI 1.42–3.49.

When we considered exposure to barium enema occurring at least 10 years before prostate cancer diagnosis, the association with increased prostate cancer risk strengthened: adjusted OR 2.49, 95% CI 1.07–5.78. Similarly, for exposures to hip/pelvic x-rays 10 or more years before diagnosis, the association remained significant even after adjustment and became stronger: OR 2.65, 95% CI 1.60–4.39.

In the case of exposures dating back at least 20 years before diagnosis, significant results were obtained only for hip/pelvic x-rays (OR 2.87 95% CI 1.47–5.62). No significant associations were found between increased prostate cancer risk and exposures to barium meal, upper leg x-rays or IVP.

### Subjects with a family history of cancer

Table 3 summarises the results of the analysis of those subjects with a family history of cancer. For exposures at least 5 years before diagnosis of prostate cancer, significantly raised odds ratios were found in the case of hip/pelvic x-rays, OR 3.46, 95% CI 1.44–8.34. Even after adjustment the odds ratios were significantly increased (OR 3.55, 95% CI 1.46–8.58). When exposures dating at least 10 years and 20 years before diagnosis were considered, only hip/pelvic x-rays were associated with a significantly increased risk of prostate cancer. After adjustment for age at diagnosis and social class, these associations remained significant: OR 5.01, 95% CI 1.64–15.31, and OR 14.23, 95% CI 1.83–110.74, respectively. No significant associations were found between exposures to any of the other diagnostic radiology procedures (barium meal, barium enema, IVP, upper leg x-rays) and prostate cancer risk.

### Aetiologic fraction

Table 4 summarises the results for aetiologic fraction. The aetiologic fraction was the highest for exposures to hip/pelvic x-rays as compared with other exposures. About 24% percent of prostate cancer in the study population was attributable to hip/pelvic x-rays exposures. When family history factor was controlled for, the aetiologic fraction decreased slightly to 21% percent.

Table 5 indicates the mean minimum and maximum exposure values estimated for the various examination types considered (with the exception of the upper leg examination where the dose to the prostate was considered to be negligible).

## DISCUSSION

The results of our case–control analysis show that exposure to some diagnostic radiological procedures may be associated with an increased risk of prostate cancer. We found a significant association between increased prostate cancer risk and exposure to a hip/pelvic x-ray irrespective of a family history of cancer. Those exposed to hip x-rays were about two and a half times more likely to have prostate cancer as compared to those without a previous history of hip x-rays (when considering exposures at least a decade prior to diagnosis). Similarly, those who had a barium enema (at least 10 years prior to diagnosis) were about two and half times more likely to have prostate cancer as compared to those not exposed to barium enemas. The aetiologic fraction calculations showed that approximately a fifth of prostate cancer cases in our study population were attributable to past hip/pelvic x-rays. For the UK the number of cases of YOPC per year is estimated at 3800 so this equates to about 760 cases of YOPC being attributable to past hip/pelvic x-rays (CRUK, 2007).

### Strengths and limitations of the study

Our study is unique in looking at the relationship between exposure to low dose ionising radiation from diagnostic radiology procedures and prostate cancer risk at young age of onset. Moreover, restricting our study to YOPC cases (i.e. below the age of 60 years) ensures that only aggressive cases of prostate cancer are considered rather than mere age-associated prostate changes.

There are some potential limitations with our study, which need discussion. We had incomplete data on dates of all exposures. To exclude a temporal bias we only included exposure data on diagnostic procedures, which could be dated in the analysis. However, we were unable to investigate the effects of cumulative exposure to radiation from diagnostic procedures in the region of the prostate on prostate cancer risk.

Another limitation is that we were unable to stratify our analysis by family history of prostate cancer because of small numbers. This would have enabled us to assess the possible role of inherited predisposition to radiation-induced prostate cancer. We, therefore conducted a stratified analysis by family history of any cancer as a proxy marker of genetic susceptibility.

We considered the possibility that the radiological procedures we have studied may have led to the diagnosis of prostate cancer. However, with the exception of IVP, upper leg x-rays and hip/pelvic x-rays it is unlikely that other procedures like barium enema, barium meal, could contribute to the diagnosis of prostate cancer. It is also unlikely that exposure is related to diagnosis as we only included exposures dating at least 5 years prior to the prostate cancer diagnosis.

Finally, as in all case–control studies, the potential problem of recall bias exists. In our study it is unlikely that cases would have preferentially recalled some radiological procedures over others. This is further illustrated by our results in which not all procedures are significantly associated with prostate cancer. Similarly, cases would not have any idea of the dose of ionising radiation delivered to the prostate from different procedures.

### Interpretation of results

To our knowledge this is the first study to report the relationship between low dose ionising radiation from diagnostic procedures and prostate cancer risk. The link between ionising radiation and cancer is well established in the literature. Most of the evidence comes from studies of atomic bomb survivors (Preston *et al*, 2003a). It has been estimated that in the UK about 0.6% of the cumulative risk of cancer to age 75 years can be attributable to diagnostic x-rays and this is equivalent to about 700 cancer cases a year (Berrington de Gonzalez and Darby, 2004).

Radiation-induced solid tumours can develop after a latency period of 10–40 years; however, shorter latent periods could be observed (Cormack *et al*, 2004). There is some debate about the lowest dose of ionising radiation that can result in increased carcinogenic risks. In the case of x-rays, there is good evidence of an increased cancer risk at acute doses greater than 50 mSv and some evidence that cancer risks increase at doses above 5 mSv (Brenner *et al*, 2003). For protracted doses, there is reasonable evidence that a lifetime exposure exceeding 50 mSv increases cancer risks (Brenner *et al*, 2003; HPS, 2004). Single diagnostic x-ray procedures deliver full body radiation effective doses in the range of 0.01–10 mSv (Hart and Wall, 2002). While we have attempted to estimate radiation doses delivered to the prostate gland in this paper (Table 5) in the absence of data on actual exposure conditions for the cases, this should be interpreted with caution. It is possible that the dose to the prostate for any individual case could have been significantly greater or smaller than these values.

In our study, while there appears to be a relationship between the increased incidence of prostate cancer with hip or pelvic examinations, no such relationship is seen with IVP examinations even though a similar dose to the prostate has been estimated for both these examinations. This could be because of the small number of IVP exposures in our study population, which affected the statistical power.

When explaining causality in epidemiological studies, biological plausibility is an important criterion to be considered. Brenner *et al* (2003) point out that based on a linear dose–response, non-threshold model, ‘the risk of radiation induced cancer should decrease linearly, without a threshold to arbitrarily low doses’ (Brenner *et al*, 2003). A pooled analysis of eight cohorts supported the linearity of the radiation dose response for breast cancer, and suggested a similarity in risks for acute and fractionated high-dose rate exposures, though with a much smaller effect from low-dose rate protracted exposures (Preston *et al*, 2002). However, as Cormack *et al* (2004) point out, it is logical to assume that qualitatively similar biological processes would be active, at different doses. In addition, The Health Physics Society states that the possibility that health effects might occur at small doses of ionising radiation should not be entirely discounted (HPS, 2004).

Further, the possible role of genetic susceptibility to radiation-induced prostate cancer should be considered. It is well established that family history of prostate cancer increases an individual's risk of developing prostate cancer (Gronberg, 2003). It has been suggested in cell studies that inherited G_2_ chromosomal radiosensitivity in few individuals is a marker for low penetrance predisposition to common cancers such as breast cancer and colorectal cancer (Baria *et al*, 2001). This indicates a subpopulation of sensitive individuals who will be exceedingly sensitive to radiation-induced cancers (Brenner *et al*, 2003). A study of cell lines from prostate cancer cases found greater G_2_ chromosomal radiosensitivity when compared with cell lines from healthy controls (Howe *et al*, 2005).

Thus there is the possibility for gene-environment interactions to take place in the case of exposures to low dose ionising radiation. We explored this by carrying out a separate analysis for a subgroup of our sample that had a family history of cancer. We had considered a separate analysis for subjects with a family history of prostate cancer *per se* but because of the small numbers in this subset this was not viable. Our results showed that even in the subset comprising subjects with a family history of cancer, prostate cancer cases were more likely to be exposed to hip/pelvic x-rays. Moreover, the exposure odds in this subgroup of cases increased when compared with the exposure odds obtained from the analysis of all subjects (irrespective of family history). Previous reports of candidate gene analyses in YOPC have shown a small proportion harbour mutations in DNA repair genes such as BRCA2 and CHEK2 (Dong *et al*, 2003; Edwards *et al*, 2003; Cybulski *et al*, 2004). There is evidence of susceptibility to breast cancers induced by low dose ionising radiation from diagnostic procedures such as chest x-rays in the case of BRCA1/2 mutation carriers (Andrieu *et al*, 2006). It could be extrapolated from these studies and our own results that certain individuals may be genetically susceptible to prostate cancers induced by exposure to low-dose ionising radiation provided by diagnostic radiological procedures in the region of the prostate gland.

Finally, it is worth noting that computed tomography (CT) is now widely used in place of many radiographic examinations and the dose to the prostate from a CT scan of the pelvic region can be around 20 mGy, which is comparable to the dose to the prostate from a barium enema examination. The International Commission on Radiological Protection (ICRP, 2007) lists the tissues or organs known to be radiation-sensitive to cancer induction. Normalised tissue weighting factors that identify the relative sensitivities of these organs and tissues is used in the calculation of effective dose. This list does not specifically identify a tissue-weighting factor for the prostate gland. The weighting factor for the prostate is shared between 13 remainder organs whose total weighting factor is 0.12. The Commission's 2007 update has reduced the judged value of the tissue-weighting factor for the gonads based on estimates of genetic risks from continuous low-dose rate exposures. One of the implications of our study results is the need for a renewed evaluation of the ICRP tissue-weighting factors to take into account the possible higher relative sensitivity of the prostate gland. Until more evidence is available, practitioners should adopt a more cautious approach to diagnostic radiology procedures and consider an increasing use of magnetic resonance imaging (MRI) as opposed to CT and better shielding of the prostate during such procedures. This is echoed in the recent ICRP recommendations which emphasise that the reduction in the gonadal tissue weighting factor does not justify allowing controllable gonadal exposures to increase in magnitude (ICRP, 2007).

## Figures and Tables

**Table 1:** Baseline demographic characteristics of cases and controls

	**Cases (*n*=431)**	**Controls (*n*=409)**	**OR (95% CI)**
Mean age at diagnosis^a^ (years)	55.11	54.42	1.05 (1.01–1.08)
			
*Social class based on occupation*			
I	33 (8.8%)	32 (8.6%)	1.00
II	140 (37.1%)	163 (43.9%)	0.83 (0.49–1.42)
IIINM	41 (10.9%)	38 (10.2%)	1.05 (0.54–2.02)
IIIM	114 (30.2%)	92 (24.8%)	1.20 (0.69–2.10)
IV	31 (8.2%)	28 (7.5%)	1.07 (0.53–2.17)
V	9 (2.4%)	6 (1.6%)	1.46 (0.46–4.56)

CI=confidence interval; OR=odds ratio.

a For controls this corresponds to matched case patient's age at first diagnosis of prostate cancer.

**Table 2:** Case–control analysis: association between exposure to diagnostic x-rays and prostate cancer

**Exposure variable**	**Number exposed (total)**	**Crude OR (95% CI)**	**Adjusted OR^a^ (95% CI)**
*Barium meal*			
>5 years	159 (678)	1.28 (0.90–1.82)	1.21 (0.84–1.73)
>10 years	138 (657)	1.25 (0.86–1.83)	1.18 (0.80–1.72)
>20 years	90 (609)	1.04 (0.66–1.62)	0.96 (0.61–1.52)
			
*Barium enema*			
>5 years	36 (666)	2.15 (1.05–4.36)^†^	2.06 (1.01–4.20)^†^
>10 years	27 (657)	2.55 (1.10–5.90)^†^	2.49 (1.07–5.78)^†^
>20 years	10 (640)	1.61 (0.45–5.76)	1.49 (0.41–5.37)
			
*IVP (kidneys)*			
>5 years	49 (687)	1.63 (0.90–2.96)	1.67 (0.92–3.03)
>10 years	37 (675)	1.51 (0.77–2.97)	1.55 (0.79–3.06)
>20 years	19 (657)	1.77 (0.69–4.55)	1.69 (0.66–4.36)
			
*Hip/pelvic x-ray*			
>5 years	97 (583)	2.20 (1.41–3.45)^†^	2.23 (1.42–3.49)^‡^
>10 years	78 (564)	2.57 (1.56–4.23)^‡^	2.65 (1.60–4.39)^‡^
>20 years	42 (528)	2.72 (1.40–5.30)^†^	2.87 (1.47–5.62)^†^
			
*Upper leg x-ray*			
>5 years	60 (643)	1.12 (0.66–1.90)	1.11 (0.65–1.89)
>10 years	48 (631)	1.46 (0.81–2.66)	1.45 (0.80–2.64)
>20 years	39 (622)	1.35 (0.70–2.60)	1.37 (0.71–2.65)

OR=Odds Ratio; CI=Confidence interval.

^†^*P*-value significant; ^‡^*P*-value<0.001.

a Adjusted for age at diagnosis and social class.

**Table 3:** Case–control analysis of subjects with a family history of cancer: association between exposure to diagnostic x-rays and prostate cancer

**Procedure**	**Number exposed (total)**	**Unadjusted OR (95% CI)**	**Adjusted OR^a^ (95% CI)**
*Barium meal*			
>5 years	83 (373)	1.17 (0.71–1.92)	1.09 (0.66–1.81)
>10 years	71 (361)	1.18 (0.70–1.99)	1.08 (0.63–1.86)
>20 years	41 (331)	0.94 (0.49–1.81)	0.86 (0.44–1.67)
			
*Barium enema*			
>5 years	22 (386)	1.74 (0.69–4.36)	1.60 (0.63–4.03)
>10 years	19 (383)	2.27 (0.80–6.44)	2.12 (0.74–6.04)
>20 years	6 (370)	0.81 (0.16–4.07)	0.68 (0.13–3.45)
			
*IVP*			
>5 years	26 (390)	1.48 (0.64–3.41)	1.44 (0.62–3.33)
>10 years	18 (382)	1.23 (0.47–3.25)	1.19 (0.45–3.16)
>20 years	10 (374)	1.83 (0.47–7.19)	1.62 (0.41–6.42)
			
*Hip/pelvic x-ray*			
>5 years	30 (299)	3.46 (1.44–8.34)^†^	3.55 (1.46–8.58)^†^
>10 years	22 (291)	4.74 (1.56–14.3)^†^	5.01 (1.64–15.31)^†^
>20 years	14 (283)	13.69 (1.77–106.16)^†^	14.23 (1.83–110.74)^†^
			
*Upper leg x-ray*			
>5 years	24 (360)	0.89 (0.39–2.04)	0.83 (0.36–1.93)
>10 years	20 (356)	1.39 (0.54–3.58)	1.32 (0.51–3.43)
>20 years	18 (354)	1.50 (0.55–4.09)	1.46 (0.53–4.02)

OR=Odds Ratio; CI=Confidence interval.

† *P*-value significant.

a Adjusted for age at diagnosis and social class.

**Table 4:** Aetiologic fractions

**Exposure**	**Aetiologic fraction^a^ (%)**	**Aetiologic fraction^b^ (%)**
Barium meal	7.8	5.8
Barium enema	10.6	4.4
IVP kidney	6.4	7.0
Hip and pelvic x-ray	23.7	21.3
Upper leg x-ray	4.9	7.8

a Not controlled for confounding.

b Controlled for family history.

**Table 5:** Mean minimum and maximum estimates of the dose to the prostate gland

**Examination**	**Mean minimum dose (mGy)^a^**	**Mean maximum dose (mGy)^a^**
Barium enema	10.0	25.0
Barium meal	0.2	0.4
IVP	3.0	4.0
HIP/pelvic	2.0	5.0

a For diagnostic x-rays the absorbed dose in mGy is numerically equal to the equivalent organ dose in mSv.
